# Three-year experience with immediate extubation in pediatric patients after congenital cardiac surgery

**DOI:** 10.1186/s13019-020-1051-3

**Published:** 2020-01-06

**Authors:** Christopher F. Tirotta, Stephen Alcos, Richard G. Lagueruela, Daria Salyakina, Weize Wang, Jessica Hughes, Marysory Irizarry, Redmond P. Burke

**Affiliations:** 1 Cardiac Anesthesia, Department of Anesthesiology, Nicklaus Children’s Hospital, Miami, USA; 2 Research Institute, Nicklaus Children’s Hospital, Miami, USA; 3 Division of Cardiovascular Surgery, Nicklaus Children’s Hospital, Miami, USA

**Keywords:** Cardiac surgery, Congenital heart disease, Early extubation, Extubation, Pediatric

## Abstract

**Background:**

In pediatric cardiac anesthesiology, there is increased focus on minimizing morbidity, ensuring optimal functional status, and using health care resources sparingly. One aspect of care that has potential to affect all of the above is postoperative mechanical ventilation. Historically, postoperative ventilation was considered a must for maintaining patient stability. Ironically, it is recognized that mechanical ventilation may increase risk of adverse outcomes in the postoperative period. Hence, many institutions have advocated for immediate extubation or early extubation after many congenital heart surgeries which was first reported decades ago.

**Methods:**

637 consecutive patient charts were reviewed for pediatric patients undergoing cardiac surgery with cardiopulmonary bypass. Patients were placed into three groups. Those that were extubated in the operating room (OR) at the conclusion of surgery (Immediate Extubation or IE), those that were extubated within six hours of admission to the ICU (Early Extubation or EE) and those that were extubated sometime after six hours (Delayed Extubation or DE). Multiple variables were then recorded to see which factors correlated with successful Immediate or Early Extubation.

**Results:**

Overall, 338 patients (53.1%) had IE), 273 (42.8%) had DE while only 26 patients (4.1%) had EE. The median age was 1174 days for the IE patients, 39 days for the DE patients, whereas 194 days for EE patients (*p* < 0.001). Weight and length were also significantly different in at least one extubation group from the other two (*p* < 0.001). The median ICU LOS was 3 and 4 days for IE and EE patients respectively, whereas it was 9.5 days for DE patients (*p* < 0.001). DE group had a significant longer median anesthesia time and cardiopulmonary bypass time than the other two extubation groups (*p* > 63,826.88 < 0.001). Regional low flow perfusion, deep hypothermia, deep hypothermic circulatory arrest, redo sternotomy, use of other sedatives, furosemide, epinephrine, vasopressin, open chest, cardiopulmonary support, pulmonary edema, syndrome, as well as difficult intubation were significantly associated with delayed extubation (IE, EE or DE).

**Conclusions:**

Immediate and early extubation was significantly associated with several factors, including patient age and size, duration of CPB, use of certain anesthetic drugs, and the amount of blood loss and blood replacement. IE can be successfully accomplished in a majority of pediatric patients undergoing surgery for congenital heart disease, including in a minority of infants.

## Background

Congenital heart disease (CHD) affects nearly 1% of – or about 40,000 – births per year in the United States [1]. About 25% of babies with a CHD have critical CHD. Infants with critical CHD generally need surgery or other procedures in their first year of life, many of these surgeries requiring cardiopulmonary bypass. Moreover, nearly all of these surgeries and procedures require general anesthesia with endotracheal intubation. Thus, the anesthesia technique plays an integral role in improving patient outcomes after congenital cardiac surgery.

In pediatric cardiac anesthesiology, there is an increased attention focused on minimizing patient trauma with emphasis on minimizing morbidity, ensuring optimal functional status, and using health care resources sparingly. One aspect of care that has potential to affect all of the above is postoperative mechanical ventilation [2, 3]. Historically, postoperative ventilation was considered a must for maintaining patient stability. Ironically, it is recognized that mechanical ventilation may increase the risk of adverse outcomes in the postoperative period [4]. Hence, many institutions have advocated for immediate extubation (IE) or early extubation (EE) after congenital heart surgeries which was first reported decades ago [5–7].

,IE/EE may translate into earlier enteral feed advancement and potentially a shorter hospital length of stay [8]. IE may also lessen the need for analgesic and sedative medications because endotracheal intubation can be a noxious stimulant. The association of prolonged endotracheal intubation with nosocomial infections, including ventilator-associated pneumonia, is well documented [3].

The rationale for conducting this study is to confirm that IE can be done safely in pediatric patients after congenital cardiac surgery with improved patient outcomes. The factors that are associated or correlated with successful IE will be analyzed; included will be both the anesthesia and non-anesthesia/surgical variables.

## Methods

After receiving Institutional Review Board (IRB) exempt status from the Research Institute of Nicklaus Children’s Hospital, we retrospectively reviewed the charts of all patients undergoing cardiac surgery for congenital heart disease between May 1, 2014 to June 30, 2017. These cases were done by one of three pediatric cardiac anesthesiologists and one of three cardiac surgeons; the cases were equally distributed between the anesthesiologists, but one surgeon performed over 90% of the surgeries. Patients were placed into three groups. Those that were extubated in the operating room (OR) at the conclusion of surgery (Immediate Extubation or IE), those that were extubated within six hours of admission to the ICU (Early Extubation or EE) and those that were extubated sometime after six hours (Delayed Extubation or DE). We then recorded the following variables:

Patient related factors like age at time of operation (days), sex of patient, weight and length (kg and cm).

Surgical related factors like duration of: cardiopulmonary bypass time (CPB), aortic cross clamp time, regional low flow perfusion time (RLF), surgical time, anesthesia time, and time between end of surgery and room out (ES-RO). These also include lowest temperature achieved during surgery, use of deep hypothermia (low temp of less than 25 °C), duration deep hypothermic circulatory arrest (DHCA), and whether the surgery entailed a redo-sternotomy.

Transfusion related factors like volume of blood products used in the OR. These include packed red blood cells (PRBC) cell saver, fresh frozen plasma (FFP), plateletpheresis, cryoprecipitate, human fibrinogen concentrate (HFC), urine output, and estimated blood loss (EBL) in first 24 h post-op.

Anesthesia and pharmacological factors like the amount of Lasix, mannitol, and crystalloid used. Also, the need for inotropic support which includes the following: epinephrine, milrinone, and vasopressin. We looked at whether the drug was used and the amount. We recorded the amount of the following anesthetics: fentanyl, morphine, propofol, midazolam, other benzodiazepines (like lorazepam), other sedatives (like ketamine), dexmedetomidine, neostigmine, and sugammadex,

And finally other factors like: open chest on leaving the OR, need for Cardiopulmonary Support (CPS) leaving OR, identity of the anesthesiologist, length of Intensive Care Unit (ICU) stay, patient length of hospital stay (PLOS), difficult intubation, reintubation within 24 h of ICU arrival, and the presence of congenital birth syndromes.

### Statistical analysis methods

Descriptive statistics were used to summarize characteristics of the study patients. Sub-group analysis was conducted among infants ≤1 year of age. Frequencies and percentages were used to present categorical variables overall and stratified by extubation group (IE [immediate extubation], EE [early extubation], and DE [delayed extubation]). Sample median and interquartile range (IQR) of variables including age, weight, length, ICU length of stay (LOS), hospital LOS, intubation days, anesthesia time, CPB time, and time of ES-RO were calculated due to non-normal distribution of the data and were reported for overall and by extubation groups. To determine if there is a significant difference between extubation groups (IE, EE, or DE), fisher exact tests and Kruskal Wallis test was used for categorical and continuous variables respectively.

In order to understand whether there was a significant difference by extubation group in procedure duration, or the dose of an administered medication/blood product during the surgery, adjusted median regression was performed for mannitol, amount, and epineprhine respectively; Zero Truncated Poisson regression was used to predict intubation days, while log-normal regression was applied to predict other continuous variables. Extubation group (IE + EE vs. DE) was used as the main predictor, while age, weight, and length were adjusted as covariates. Patients with IE and EE were included as one group due to small sample size in the EE group. Blood products were measured at two times. Thus, generalized linear models with repeated measures was used to assess the effect of extubation group on the dose of each blood product overtime adjusting for age, weight, and length as covariates. For the administered medications/blood products and procedures, not every patient received these items. Thus, when we compare the doses of each medication/blood product, or duration of the procedure by the extubation groups, patients that did not receive the product/procedure were excluded from the log-normal regression and generalized linear models. For example, only 536 out of the 637 patients had their aorta cross clamped (XC) as part of the procedure; we conducted the regression analysis using the duration of XC from the 536 patients as the outcome.

Statistical analyses were performed by using the statistical software package SAS Enterprise Guide 7.1 (SAS Institute Inc., Cary, NC). All statistical analyses were performed at 0.05 level of significance.

## Results

Six hundred thirty-seven cases are included in this analysis (Table 1). Overall, 338 patients (53.1%) had IE, 273 (42.8%) had DE, while only 26 patients (4.1%) had EE. The median age was 1174 days for the IE patients, 39 days for the DE patients, whereas 194 days for EE patients (*p* < 0.001). Weight and length were also significantly different in at least one extubation group from the other two (*p* < 0.001). The median ICU LOS was three and four days for IE and EE patients respectively, whereas it was 9.5 days for DE patients (p < 0.001). DE group had a significant longer median anesthesia time and CPB time in minutes than the other two extubation groups (p < 0.001). Median time for End of Surgery to Room Out (ES-RO) for EE was 16.5 min, whereas it was 14 min in both IE and DE groups (*p* = 0.029). RLF, deep hypothermia, DHCA, redo, use of other sedatives, lasix, epinephrine, vasopressin, open chest, CPS, pulmonary edema, syndrome, as well as difficult intubation were significantly associated with extubation time (IE, EE or DE) (Table 2, *p* < 0.05).

**Table 1:** Patient characteristic and times

Variable	Overall		Extubation						p^b^
			IE		DE		EE		
	N	Median (Interquartile Range)	N	Median (Interquartile Range)	N	Median (Interquartile Range)	N	Median (Interquartile Range)	
Age (days)	637	236 (1545)	338	1174 (3193)	273	39 (181)	26	194 (1098)	**< 0.001**
Weight (kg)	637	7.1 (12.6)	338	14 (24)	273	3.5 (2.9)	26	7.5 (8.8)	**< 0.001**
Length (cm)	634	67 (50)	338	96 (65)	270	52 (13)	26	65 (36)	**< 0.001**
ICU LOS (days)	635	5 (7)	337	3 (4)	272	9.5 (15.0)	26	4 (5)	**< 0.001**
PLOS (days)	634	8 (10)	337	5 (4)	271	12 (16)	26	5.5 (5)	**< 0.001**
Anes time (min)	637	262 (110)	338	247 (90)	273	298 (117)	26	230 (111)	**< 0.001**
CPB (min)	635	99 (73)	337	84 (44)	272	138 (94)	26	71 (31)	**< 0.001**
Time (ES-RO) (min)	635	14 (7)	337	14 (8)	272	14 (7)	26	17 (8)	**0.029**

^b^*P* values were determined using Fisher’s exact tests for categorical variables and Kruskal Wallis tests for continuous variables. *P* < 0.05 were in bold character

**Table 2:** Patients characteristics, medical conditions, and therapies (*N* = 637)

Variable	Overall	Extubation Time			p^b^
	(*N* = 637)	IE (*N* = 338)	DE (*N* = 273)	EE (*N* = 26)	
	N (%^a^)	N (%^a^)	N (%^a^)	N (%^a^)	
Sex					0.584
Male	349 (54.8)	184 (54.4)	153 (56.0)	12 (46.2)	
Female	285 (44.7)	153 (45.3)	118 (43.2)	14 (53.8)	
Regional Low Perfusion					**< 0.001**
Yes	92 (14.4)	4 (1.2)	87 (31.9)	1 (3.8)	
No	544 (85.4)	334 (98.8)	185 (67.8)	25 (96.2)	
Deep Hypothermia (Temp < 25)					**< 0.001**
Yes	150 (23.5)	14 (4.1)	134 (49.1)	2 (7.7)	
No	487 (76.5)	324 (95.9)	139 (50.9)	24 (92.3)	
DHCA					**< 0.001**
Yes	42 (6.6)	2 9 (0.6)	40 (14.7)	0 (0.0)	
No	595 (93.4)	336 (99.4)	233 (85.3)	26 (100.0)	
Redo sternotomy					**< 0.001**
Yes	188 (29.5)	135 (39.9)	45 (16.5)	8 (30.8)	
No	446 (70.0)	201 (59.5)	228 (83.5)	17 (65.4)	
Other Benzodiazepines					
No	636 (99.9)	337 (99.7)	273 (100.0)	26 (100.0)	NA
Other Sedatives					**0.004**
Yes	45 (7.1)	14 (4.1)	30 (11.0)	1 (3.8)	
No	591 (92.8)	324 (95.9)	242 (88.6)	25 (96.2)	
Lasix					**0.004**
Yes	563 (88.4)	310 (91.7)	230 (84.2)	23 (88.5)	
No	43 (6.8)	13 (3.8)_	29 (10.6)	1 (3.8)	
Factor VII					NA
No	637 (99.5)	338 (100)	273 (100.0)	26 (100.0)	
Milrinone					0.689
Yes	633 (99.4)	335 (99.1)	272 (99.6)	26 (100.0)	
No	4 (0.6)	3 (0.9)	1 (0.4)	0 (0.0)	
Epinephrine					**< 0.001**
Yes	155 (24.3)	8 (2.4)	144 (52.7)	3	
No	482 (75.7)	330 (97.6)	129 (47.3)	23	
Vasopresin					**0.008**
Yes	7 (1.1)	0 (0.0)	7 (2.6)	0 (0.0)	
No	630 (98.9)	338 (100.0)	266 (97.4)	26 (100.0)	
Open chest					**< 0.001**
Yes	108 (17.0)	0 (0.0)	108 (39.6)	0 (0.0)	
No	529 (83)	338 (100.0)	165 (60.4)	26 (100.0)	
Cardiopulmonary Support					**< 0.001**
Yes	20 (3.1)	0 (0.0)	20 (7.3)	0 (0.0)	
No	617 (96.9)	338 (100.0)	253 (92.7)	26 (100.0)	
Pulmonary Edema					**0.001**
Yes	13 (2.0)	1 (0.3)	12 (4.4)	0 (0.0)	
No	624 (98.0)	337 (99.7)	261 (95.6)	26 (100.0)	
Reintubation within 24 h					0.100
Yes	17 (2.7)	5 (1.5)	11 (4.0)	1 (3.8)	
No	617 (96.9)	331 (97.9)	261 (95.6)	25 (96.2)	
Syndrome					**< 0.001**
Yes	113 (17.7)	34 (10.1)	74 (27.1)	5 (19.2)	
No	519 (81.5)	302 (89.3)	196 (71.8)	21 (80.8)	
Difficult intubation					**0.001**
Yes	13 (2.0)	1 (0.3)	12	0 (0.0)	
No	618 (97.0)	333 (98.5)	259 (94.9)	26 (100.0)	

Note. ^a^%s are column percentages except the first row. The sum of %s of a variable may not be 100 due to missing values

^b^*P* values were determined using Fisher’s exact tests for categorical variables and Kruskal Wallis tests for continuous variables. *P* < 0.05 were in bold character

Results suggest a significant an association between extubation group and aortic cross clamp time, the longer the cross-clamp time the more likely the patient would be in the DE group, controlled by patient’s age, weight and length (Table 3, *p* < 0.001). Significant association was also found between extubation group with low temperature, urine output, doses of fentanyl, midazolam, rocuronium, human fibrinogen concentrate (HFC), milrinone, epinephrine, and ICU EBL, adjusting for patient’s age, weight and length (Table 3, *p* < 0.05).

**Table 3:** Descriptive statistics of procedures and administered medications with results of regression analyses

Variable^a^	Underwent Treatment/Procedure		Patients Underwent the Treatment/Procedure						
	Yes	No	Overall		Delayed Extubation		Immediate/Early Extubation		
	N (%)	N (%)	N	Median (Qrange)	N	Median (Qrange)	N	Median (Qrange)	p^b^
Aortic cross clamp (min)	536 (84)	101 (16)	536	58.4 (48.0)	249	77.0 (62.0)	287	50.0 (33.0)	**< 0.001**
Low Temperature (°C)	630 (98.9)	7 (1.1)	630	29.1 (6.5)	270	24.0 (12.0)	360	30.6 (4.50)	**< 0.001**
DHCA (min)	42 (6.6)	5.95 (93.4)	42	25 (33.0)	40	21.0 (33.5)	2	26.5 (1.00)	0.662
Urine (cc/kg)	616 (96.7)	21 (3.3)	616	9.51 (14.2)	254	6.47 (9.74)	362	12.7 (16.3)	**0.024**
Fentanyl (mcg/kg)	606 (95.1)	31 (4.9)	606	10.26 (16.6)	269	22.7 (18.9)	337	5.32 (5.44)	**< 0.001**
Morphine (mg/kg)	42 (6.6)	595 (93.4)	42	0.22 (0.16)	7	0.22 (0.23)	35	0.23	NA
Propofol (mg/kg)	478 (75.0)	159 (25.0)	478	2.82 (3.2)	176	2.86 (3.85)	302	2.81 (2.75)	0.875
Dexmedetomidine (mcg/kg)	586 (92)	51 (8.0)	586	2.88 (2.3)	229	3.03 (2.58)	357	2.80 (1.99)	0.240
Rocuronium (mg/kg)	636 (99.8)	1 (0.2)	636	3.13 (2.0)	272	4.07 (2.60)	364	2.72 (1.34)	**0.019**
Neostigmine (mg/kg)	277 (43.5)	360 (56.5)	277	0.07 (0.01)	21	0.07 (0.02)	256	0.07 (0.01)	0.653
Sugammadex (mg/kg)	97 (15.2)	540 (84.8)	97	4.35 (2.2)	7	4.35 (1.02)	90	4.37 (2.34)	0.079
Midazolam (mg/kg)	153 (24.0)	484 (76.0)	153	0.09 (0.1)	29	0.20 (0.25)	124	0.08 (0.09)	**< 0.001**
Other Sedatives (mg/kg)	48 (7.5)	589 (92.5)	48	6.48 (10.8)	31	4.63 (3.27)	17	15.0 (0.79)	0.242
Mannitol (mg/kg)	405 (63.6)	232 (36.4)	405	400 (109.0)	165	406. (100.)	240	400 (213)	0.298
Lasix (mg/kg)	564 (88.5)	73 (11.5)	564	0.42 (0.4)	231	0.59 (0.32)	333	0.31 (0.25)	0.997
NS Amount (cc/kg)	631 (99.1)	6 (0.9)	631	17.77 (16.8)	268	20.6 (15.9)	363	16.2 (15.6)	0.669
HFC (mg/kg)	400 (62.8)	237 (37.2)	400	71.1 (70.0)	222	136. (74.7)	178	70.0 (3.97)	**0.008**
Milrinone (mg/kg)	635 (99.7)	2(0.3)	635	0.15 (0.04)	273	0.16 (0.06)	362	0.14 (0.03)	**< 0.001**
Epinephrine (mg/kg)	1.57 (24.7)	480 (75.4)	157	0.01 (0.01)	146	0.01 (0.01)	11	0.00 (0.01)	**0.046**
Vasopresin (mcg/kg)	7 (1.1)	630 (98.9)	7	0.08 (0.14)	7	0.08 (0.14)	0	NA	NA
ICU EBL (cc/kg)	612 (96.1)	25 (3.9)	612	21.5 (28.5)	260	31.0 (43.5)	352	15.0 (19.0)	**0.026**
Intubation (days)^#^	296 (46.5)	341 (53.5)	632	0 (3.0)	270	3.00 (4.00)	362	0.00 (0.00)	< 0.001

Note. ^a^ 0 s in variables are excluded. # 0 s were NOT Excluded (Intubation Days)

^b^*P*-values are determined from median regression for Mannitol, Amount, and Epi; Poisson regression for Intubation days, and Log-Normal regression for other continuous variables. Extubation group (IE + EE vs. DE) was used as the main predictor adjusting for age, weight, and length as covariates. NAs are due to limited sample size. *P* < 0.05 are in bold character

On average, a significant association was found between extubation group and the dose of PRBC in that patients with delayed extubation had a higher dose of PRBC (*p* < 0.001), controlling for age, weight and length. There was no significant difference in the amount of any other blood product.

Overall, of the total 637 patients, 350 (54.9%) patients were infants (Table 4). Of the 350 patients, 232 (66.3%) had DE, 103 (29.4%) had IE, and 14 (4.3%) had EE. The median age was 20 days for the DE patients, 174 days for the IE patients, and 157 days for EE patients (*p* < 0.0001). Weight and length were also significantly different in at least one extubation group from the other two (p < 0.0001). The youngest patient to successfully undergo IE was four days old and the smallest was 3.3 kg. The median ICU LOS was 4 and 5 days for IE and EE patients respectively, whereas it was 11 days for DE patients (*p* < 0.0001). When compared to IE and EE, DE group had a significant longer median anesthesia time (285 vs 235[IE], 228[EE] minutes) and CPB time (134 vs 80 [IE &EE] minutes) (*p* > 0.0001). Median time (ES-RO) was 14 min for DE patients, 15 min for IE patients, and 18 min for EE patients (*p* = 0.0126). RLP, deep hypothermmia, DHCA, redo sternotomy, use of other sedatives, epinephrine, open chest, and CPS were significantly associated with extubation time (IE, EE or DE) (Table 5, *p* < 0.05).

**Table 4:** Patient characteristic and times

Variable	Overall		Extubation						p^b^
			DE		IE		EE		
	N	Median (Interquartile Range)	N	Median (Interquartile Range)	N	Median (Interquartile Range)	N	Median (Interquartile Range)	
Age (days)	350	94 (164.0)	232	20 (97.0)	103	174 (82.0)	15	157 (38.0)	**< 0.0001**
Weight (kg)	350	4.45 (3.0)	232	3.4 (1.62)	103	6.3 (1.6)	15	6.4 (1.7)	**< 0.0001**
Length (cm)	347	56 (14.0)	229	51 (9.0)	103	64 (6.5)	15	62 (4.0)	**< 0.0001**
ICU LOS (days)	348	8 (11.0)	231	11 (15.0)	102	4 (4.0)	15	5 (6.0)	**< 0.0001**
PLOS (days)	347	9 (14.0)	230	13 (18.0)	102	6 (4.0)	15	6 (5.0)	**< 0.0001**
Intubation (days)	346	2 (4.0)	229	3 (3.0)	102	0 (0.0)	15	4 (4.0)	**< 0.0001**
Anes time (min)	350	258.5 (99.0)	232	285 (111.5)	103	235 (56.0)	15	228 (72.0)	**< 0.0001**
CPB (min)	350	104 (74.0)	232	134 (88.5)	103	80 (30.0)	15	80 (28.0)	**< 0.0001**
Time (ES-RO)(min)	348	14 (8.0)	231	14 (7.0)	102	15 (7.0)	15	18 (9.0)	**0.0126**

^b^ *P*-values are determined using Fisher’s exact tests for categorical variables and Kruskal Wallis tests for continuous variables. *P* < 0.05 are in bold character. NAs are due to limited sample size. *P* < 0.05 are in bold character

**Table 5:** Patients characteristics, medical conditions, and therapies (*N* = 350)

Variable	Overall (*N* = 350)	Extubation Time			p^b^
		DE (*N* = 232)	IE (*N* = 103)	EE (*N* = 15)	
	N (%^a^)	N (%^a^)	N (%^a^)	N (%^a^)	
Sex					0.2433
Male	206 (58.9)	136 (58.6)	64 (62.1)	6 (40.0)	
Female	141 (40.3)	94 (40.5)	38 (36.9)	9 (60.0)	
Regional Low Perfusion					**< 0.0001**
Yes	88 (25.1)	85 (36.6)	3 (2.9)	.	
No	261 (74.6)	146 (62.9)	100 (97.1)	15 (100.0)	
Deep Hypothermia (Temp < 25)					**< 0.0001**
Yes	127 (36.3)	122 (52.6)	4 (3.9)	1 (6.7)	
No	223 (63.7)	110 (47.4)	99 (96.1)	14 (93.3)	
DHCA					**< 0.0001**
Yes	42 (12.0)	40 (17.2)	2 (1.9)	.	
No	308 (88.0)	192 (82.8)	101 (98.1)	15 (100.0)	
Redo sternotomy					**< 0.0001**
Yes	66 (18.9)	27 (11.6)	34 (33.0)	5 (33.3)	
No	282 (80.6)	205 (88.4)	68 (66.0)	9 (60.0)	
Other Benzodiasepines					NA
No	350 (100.0)	232 (100.0)	103 (100.0)	15 (100.0)	
Other Sedatives					**0.0026**
Yes	30 (8.6)	28 (12.1)	2 (1.9)	.	
No	319 (91.1)	203 (87.5)	101 (98.1)	15 (100.0)	
Lasix					0.1274
Yes	33 (9.4)	27 (11.6)	5 (4.9)	1 (6.7)	
No	20 (5.7)	11 (4.7)	7 (6.8)	2 (13.3)	
Factor VII					NA
No	350 (100.0)	232 (100.0)	103 (100.0)	15 (100.0)	
Milrinone					0.5613
Yes	348 (99.4)	231 (99.6)	102 (99.0)	15 (100.0)	
No	2 (0.6)	1 (0.4)	1 (1.0)	.	
Epinephrine					**< 0.0001**
Yes	135 (38.6)	132 (56.9)	1 (1.0)	2 (13.3)	
No	215 (61.4)	100 (43.1)	102 (99.0)	13 (86.7)	
Vasopresin					0.3729
Yes	6 (1.7)	6 (2.6)			
No	344 (98.3)	226 (97.4)	103 (100.0)	15 (100.0)	
Open chest					**< 0.0001**
Yes	101 (28.9)	101 (43.5)			
No	249 (71.1)	131 (56.5)	103 (100.0)	15 (100.0)	
Cardiopulmonary support					**0.0023**
Yes	19 (5.4)	19 (8.2)			
No	331 (94.6)	213 (91.8)	103 (100.0)	15 (100.0)	
Pulmonary Edema					0.1812
Yes	13 (3.7)	12 (5.2)	1 (1.0)	.	
No	337 (96.3)	220 (94.8)	102 (99.0)	15 (100.0)	
Reintubation within 24 h					1.000
Yes	12 (3.4)	9 (3.9)	3 (2.9)	.	
No	336 (96.0)	222 (95.7)	99 (96.1)	15 (100.0)	
Syndrome					0.1183
Yes	76 (21.7)	58 (25.0)	16 (15.5)	2 (13.3)	
No	270 (77.1)	171 (73.7)	86 (83.5)	13 (86.7)	
Difficult intubation					0.0883
Yes	10 (2.9)	10 (4.3)			
No	336 (96.0)	220 (94.8)	101 (98.1)	15 (100.0)	

Note. ^a^ %s are column percentages except the first row. The sum of %s of a variable may not be 100 due to missing values

^b^ *P*-values are determined using Fisher’s exact tests for categorical and Kruskal Wallis tests for continuous variables. *P* < 0.05 are in bold character. NAs are due to limited sample size. *P* < 0.05 are in bold character

Adjusting for patient’s age, weight, and length suggested that there was a significant association observed between extubation group and XC, with longer XC times correlated with the DE group (Table 6, *p* < 0.0001). Similarly, significant association was found between extubation groups with low temperature, urine output, doses of fentanyl, dexmedetomidine, lasix, and crystalloid amount, adjusting for patient’s age, weight and length (Table 6, *p* < 0.05).

**Table 6:** Descriptive statistics of procedures and administered medications with results of regression analyses

Variable^a^	Underwent Treatment/Procedure		Patients Underwent the Treatment/Procedure						p^b^
	Yes	No	Overall		Delayed Extubation		Immediate/Early Extubation		
	N (%)	N (%)	N	Median (Qrange)	N	Median (Qrange)	N	Median (Qrange)	
Aortic cross clamp (min)	293 (83.7)	57 (16.3)	293	62.0 (52.5)	211	72.0 (60.9)	82		**< 0.0001**
Low Temperature (°C)	346 (98.9)	4 (1.1)	346	28.3 (13.3)	230	18.9 (12.0)	116	30.2 (4.9)	**< 0.0001**
DHCA (min)	42 (12.0)	308 (88.0)	42	25.0 (33.0)	40	21.0 (33.5)	2	26.5 (1.00)	0.662
Urine (cc/kg)	330 (94.3)	20 (5.7)	330	5.7 (8.6)	214	5.1 (7.6)	116	7.43 (10.3)	**0.002**
Fentanyl (mcg/kg)	347 (99.1)	3 (0.9)	347	17.9 (21.7)	232	27.0 (17.4)	115	8.82 (10.7)	**< 0.0001**
Morphine (mg/kg)	4 (1.1)	346 (98.9)	4	0.4 (2.0)	1	0.16 (0.00)	3	0.56 (3.60)	NA
Propofol (mg/kg)	246 (70.3)	104 (29.7)	246	3.0 (3.6)	150	2.8 (4.27)	96	3.08 (3.0)	0.594
Dexmedetomidine (mcg/kg)	302 (86.3)	48 (13.7)	302	3.0 (2.5)	188	2.9 (2.70)	114	3.33 (2.2)	**0.005**
Roc uranium (mg/kg)	349 (99.7)	1 (0.3)	349	3.5 (2.6)	231	4.2 (2.7)	118	2.73 (1.0)	0.742
Neostigmine (mg/kg)	94 (26.9)	256 (73.1)	94	0.01 (0.01)	13	0.1 (0.04)	81	0.07 (0.0)	0.944
Sugammadex (mg/kg)	35 (10.0)	315 (90.0)	35	5.0 (4.1)	5	4.4 (1.00)	30	5.37 (4.1)	NA
Midazolam (mg/kg)	21 (6.0)	329 (94.0)	21	0.3 (0.1)	12	0.3 (0.41)	9	0.33 (0.1)	0.676
Other Sedatives (mg/kg)	32 (9.1)	318 (90.9)	32	4.7 (3.2)	29	4.6 (2.4)	3	10.0 (18.0)	NA
Mannitol (mg/kg)	218 (62.3)	132 (37.7)	218	407.2 (100.0)	148	409.0 (100.0)	70	406.0 (100.0)	0.661
Lasix (mg/kg)	298 (85.1)	52 (14.9)	298	0.5 (0.4)	195	0.6 (0.29)	103	0.3 (0.2)	**0.008**
NS Amount (cc/kg)	344 (98.3)	6 (1.7)	344	20.0 (14.6)	227	21.4 (15.9)	117	17.5 (11.5)	**0.003**
HFC (mg/kg)	300 (85.7)	50 (14.3)	300	98.7 (71.0)	208	137.0 (75.8)	92	70.1 (55.5)	0.129
Milrinone (mg/kg)	349 (99.7)	1 (0.3)	349	0.2 (0.1)	232	0.2 (0.06)	117	0.14 (0.03)	0.103
Epinephrine (mg/kg)	135 (38.6)	215 (61.4)	135	0.01 (0.01)	132	0.01 (0.01)	3	0.00 (0.02)	0.791
Vasopresin (mcg/kg)	6 (1.7)	344 (98.3)	6	0.0 (0.1)	6	0.1 (0.1)	0	0.00	NA
ICU EBL (cc/kg)	331 (94.6)	19 (5.4)	331	30.0 (35.0)	219	38.0 (47.0)	112	24.0 (16.5)	0.858
Intubation (days)^#^	245 (70.0)	105 (30.0)	346	2.0 (4.0)	229	3.0 (3.00)	117	0.0 (0.0)	< 0.0001

Note. ^a^ 0 s in variables are excluded. # 0 s were NOT Excluded (Intubation Days)

^b^ *P*-values are determined from median regression for Mannitol, Amount, and Epi; Poisson regression for Intubation days, and Log-Normal regression for other continuous variables. Extubation group (IE + EE vs. DE) was used as the main predictor adjusting for age, weight, and length as covariates.NAs are due to limited sample size. *P* < 0.05 are in bold character

On average, a significant association was found between extubation group and the dose of PRBC in that patients with delayed extubation had a higher dose of PRBC (*p* = 0.006), controlling for age, weight and length. There was no significant difference in the amount of any other blood product.

The case mix for the IE/EE groups and the DE group are listed in Tables 7 and 8.

**Table 7:** Case Types

Case types for IE and EE	Number
Atrial septal defect repair	65
Ventricular septal defect repair	58
Fontan	43
Bidirectional cavopulmonary anastomosis	41
Right ventricular outflow tract reconstruction (pulmonary valve replacement)	40
Tetralogy of Fallot repair	30
Repair atrioventricular canal, partial or complete	18
Pulmonary artery reconstruction	12
Sub-aortic membrane resection	11
Mitral valvuloplasty or replacement	10
Repair anomalous coronary artery	9
Repair Anomalous pulmonary venous return, total or partial	4
Central shunt or Blalock-Taussig shunt	4
Aortic Valve Replacement	3
Tricuspid valvuloplasty	3
Double chambered right ventricle repair	3
Aortic arch reconstruction	2
Pulmonary artery banding	2
Rastelli procedure	1
Septal myomectomy	1
Repair Cor triatriatum	1
Ross-Konno	1
DORV repair	1

**Table 8:** Case Types

Case Types for DE	Number
Tetralogy of Fallot repair	31
Ventricular septal defect repair	25
Aortic arch reconstruction	24
Arterial switch operation	23
Stage 1 Norwood	21
Central shunt or Blalock-Taussig shunt	21
Repair anomalous pulmonary venous return, total or partial	14
Repair atrioventricular canal	13
Damus-Kaye-Stansel procedure	12
Right ventricular outflow tract reconstruction (pulmonary valve replacement)	11
Mitral valvuloplasty or replacement	10
Bidirectional cavopulmonary anastamosis	10
Truncus arteriosus repair	8
Unifocalization	8
Double outlet right veneticle repair	5
Pulmonary artery reconstruction	4
Aortic valve replacement	4
Repair anomalous coronary artery	4
Ross-Konno	2
Tricuspid valvuloplasty	2
Fontan	2
Atrial septectomy	1
Sub-aortic membrane resection	1

## Discussion

Our retrospective analysis demonstrated that a majority of older infants and pediatric congenital heart surgery patients can be successfully extubated in the operating room at the conclusion of surgery. The reintubation rate is less than 3% (2.7%) in all patients and the IE group actually had a lower reintubation rate (1.5%) than the EE (3.8%) or DE (4.0%) groups that were extubated by the ICU staff hours or days after surgery. The reintubation rate for the infants was slightly higher, but still less than 4%. There was no statistical difference between the groups. This is much better than the reported 11% (range 5 to 22%) reintubation rate in the Pediatric Cardiac Critical Care Consortium multicenter study of neonates after cardiac surgery [9] who were extubated in the ICU hours or days later. However, our data was not restricted to neonates, who have the lowest IE/EE rate of all the patients studied.

Other relevant clinical studies have shown that most children undergoing congenital heart surgery can be extubated in the operating room. Many neonates, including those undergoing complex procedures, can be extubated within the first 24 h after surgery. Early extubation (EE) has been associated with low morbidity rates and shorter lengths of intensive care unit and hospital stays [10]. In additional studies, immediate extubation was associated with a shorter Intensive Care Unit (ICU) length of stay (LOS), lower postoperative ICU costs, and minimal increase in operating room turnover time, but without an increase in reintubation rates [6]. Even though costs were not analyzed in this review, our data also demonstrated reduced LOS in the CICU and LOS in the hospital without an increase in reintubation rates. Low gestational age, preoperative ventilatory support requirement, and prolonged cardiopulmonary bypass time were inversely associated with the ability to accomplish IE [11, 12]. In a five-year cohort study of infants post repair for transposition of great arteries, greater CPB and cross-clamp times and minimum temperatures less than or equal to 30.4 degrees centigrade were associated with a lesser likelihood of IE [13]. Another five-year study demonstrated an inverse relationship between age and CPB time and early extubation [14].

Our analysis corroborates the findings listed above. The patients who experienced IE or EE had a much lower LOS in the ICU and the hospital. There was also a strong inverse relationship between younger age, smaller size, longer CPB time, longer aortic cross clamp time, use of deep hypothermia, longer RLP time, DHCA, need for epinephrine or vasopressin, ICU blood loss and the ability to achieve IE or EE. These variables are also markers for more complex repairs, which is more likely to lead to inadequate hemostasis or hemodynamic instability. Higher doses of fentanyl and midazolam were also inversely correlated with IE/EE.

The two main criteria our institution utilizes for IE is hemodynamic stability, without the need for escalating doses of inotropic support and minimal blood loss (less than 10 cc/kg/hr). Many pediatric patients undergoing cardiac surgery for congenital heart disease fit this criterion, especially patients over age one. Our standard protocol is to wean from CPB with milrinone; epinephrine is used if this is inadequate. Our data indicates that the need and dose of epinephrine is greater in the DE group. With respect to blood loss, there is a significantly greater amount in the DE group. With respect to transfusion requirements, the only difference between the groups was the amount of PRBC. The differences in the amount of other component blood products was not significant, which was surprising to the authors. It is noteworthy, that inadequate hemostasis is one of the main reasons patients at our institution do not get extubated in the OR.

Over half the patients in our analysis were infants and they represented the majority of the patients in the DE group. However, even one third of this cohort were in the IE/EE groups, including patients undergoing complex repairs. If hemodynamic stability and adequate hemostasis can be achieved in this cohort, they can be candidates for IE. The median age of the patients in this infant DE group was 20 days, significantly different than the roughly six months ages of the IE/EE cohort. However, even neonates experienced IE or EE in our data set, but we did not do a sub-analysis of neonates alone since this cohort was too small. Other investigators have also demonstrated this association between age and the ability to IE or EE. In a retrospective review of over 900 pediatric patients undergoing surgery for congenital heart disease, there was an inverse relationship between patient age and the ability to achieve EE, i.e., the younger the patient the less likely they were to experience EE [15]. Other investigators have reported similar results [16].

One noteworthy meta-analysis demonstrated that various investigators define early extubation differently [17]. Early extubation ranged from in the operating room to as much as 24 h after surgery. The anesthetic protocols also varied greatly between the studies, with varying inhalational agents, drugs and doses. Modern, short-acting anesthetics, like remifentanil, dexmedetomidine and propofol and various regional techniques, like intrathecal or caudal morphine have made early and immediate extubation possible, even for longer surgeries involving complex repairs [18]. Sugammadex has been another noteworthy addition to the anesthetic armamentarium, but more studies need to be conducted in this patient population.

Our institutional anesthetic protocol is as follows: Induction is by mask with sevoflurane if no indwelling IV is present and usually with Propofol (1–2 mg/kg) if an indwelling IV catheter is present. Etomidate or fentanyl is substituted for propofol if the physiology dictates that myocardial depression or vasodilatation should be avoided. Maintenance is with dexmedetomidine (0.5–1 mcg/kg/hr), propofol (50–100 mcg/kg/min), rocuronium and sevoflurane or some combination, coupled with the ONQ Pain Buster and more recently liposomal bupivacaine for post-operative analgesia (4 mg/kg liposomal bupivacaine admixed with 3 mL 0.25% bupivacaine for every mL of liposomal bupivacaine, plus 0.9% NS to produce a volume of 10 mL per linear inch of incision plus 10 mL for every chest tube site). Sugammadex is now routinely used for reversal of the neuromuscular block from rocuronium. Fentanyl doses are routinely kept below 5 mcg/kg in those patients where IE/EE is to be attempted; higher doses of fentanyl greatly reduce the possibility of IE/EE. Adequacy of reversal of neuromuscular blockage is assessed with a peripheral nerve stimulator at the conclusion of the case. Patients are allowed to breath spontaneously and extubation occurs when the breathing pattern is regular and tidal volumes exceed 5 mL/kg. The airway is assessed for obstruction before leaving the OR. If airway obstruction exists it is treated with airway positioning or a nasal airway. A ROTEM based transfusion protocol is utilized for hemostasis; the largest reason for delayed extubation is inadequate hemostasis.

## Conclusions

Older and larger patients were more likely to experience IE. Length of cardiopulmonary bypass, aortic cross-clamp time, use of deep hypothermia, and length of regional low flow perfusion were inversely correlated with the incidence of IE. The need for epinephrine or inotropes other than milrinone were inversely correlated with IE. Greater blood loss and need for transfusion with packed red blood cells was inversely associated with IE. IE can be successfully accomplished in a majority of pediatric patients undergoing surgery for congenital heart disease, including in a minority of infants.

## Data Availability

The datasets used and/or analyzed during the current study are available from the corresponding author on reasonable request.

## Abbreviations

CHD: Congenital heart disease
CPB: Cardiopulmonary bypass
CPS: Cardiopulmonary support
Cryo: Cryoprecipitate
CS: Cell saver
DE: Delayed extubation
DHCA: Deep hypothermic circulatory arrest
EBL: Estimated blood loss
EE: Early extubation
ES-RO: End of surgery to room out
FFP: Fresh frozen plasma
HFC: Human fibrinogen concentrate
ICU: Intensive care unit
IE: Immediate extubation
IQR: Interquartile range
IRB: Institutional review board
LOS: length of stay
OR: Operating room
Plat: Platelets
PRBC: Packed red blood cells
RLF: Regional low flow

## References

[1] Hoffman JL, Kaplan S (2002). The incidence of congenital heart disease. J Am Coll Cardiol.

[2] Robinson A (2002). Early extubation after pediatric heart surgery: the future?. Crit Care Med.

[3] Prakanrattana U, Valairucha S, Sriyoschati S, Pornvilawan S, Phanchaipetch T (1997). Early extubation following open heart surgery in pediatric patients with congenital heart diseases. J Med Assoc Thail.

[4] Fischer JE, Allen P, Fanconi S (2000). Delay of extubation in neonates and children after cardiac surgery: impact of ventilator-associated pneumonia. Intensive Care Med.

[5] Schuller JL, Bovill JG, Nijveld A, Patrick R, Marcelletti C (1984). Early Extubation of the trachea after open heart surgery for congenital heart disease. Br J Anaesth.

[6] Barash PG, Lescovich F, Katz JD, Talner NS, Stansel HC (1980). Early extubation following pediatric cardiothoracic operation: a viable alternative. Ann Thorac Surg.

[7] Heard GG, Lamberti JJ, Park SM, Waldman JD, Waldman J (1985). Early Extubation after surgical repair of congenital heart disease. Crit Care Med.

[8] Heinle JS, Fox LS (1998). Early extubation of neonates and young infants after cardiac surgery. Semin Thorac Cardiovasc Surg Pediatr Card Annu.

[9] Bennyworth BD, Mastropietro CW, Graham EM, Klugman D, Costello JM, Zhang W, Gaies M (2017). Variation in extubation failure rates after neonatal congenital heart surgery across Pediatric Cardiac Critical Care Consortium hospital. J Thorac Cardiovasc Surg.

[10] Harris KC, Holowachuk S, Pitfield S, Sanatani S, Froese N, Potts JE, Gandhi SK (2014). Should early extubation be the goal for children after congenital cardiac surgery?. J Thorac Cardiovasc Surg.

[11] Varghese J, Kutty S, Abdullah I, Hall S, Shostrom V, Hammel J (2016). Preoperative and intraoperative predictive factors of immediate extubation after neonatal cardiac surgery. Ann Thorac Surg.

[12] Mahle WT, Jacobs JP, Jacobs ML, Kim S, Kirshbom PM, Pasquali SK, Austin EH, Kanter KR, Nicolson SC, Hill KD (2016). Early extubation after repair of tetralogy of fallot and the fontan procedure: an analysis of the society of thoracic surgeons congenital heart surgery database. Ann Thorac Surg.

[13] Varghese J, Kutty S, Moukagna KSB, Craft M, Abdullah I, Hammel JM (2017). Five-year experience with immediate extubation after arterial switch operations for transposition of great arteries. Euro J Cardio Thorac Surg.

[14] Halimic M, Dinarevic SM, Begic Z, Kadic A, Pandur S, Omerbasic E (2014). Early extubation after congenital heart surgery. J Health Sci.

[15] Neirotti RA, Jones D, Hackbarth R, Paxson-Fosse G (2002). Early extubation in congenital heart surgery. Heart Lung Circ.

[16] Davis S, Worley S, Mee RBB, Harrison AM (2004). Factors associated with early extubation after cardiac surgery in young children. Pediatr Crit Care Med.

[17] Alghamdi AA, Singh SK, Hamilton BCS, Mus M, Yadava M, Holtby H, Van Arsdell GS, Al-Radi OO (2010). Early extubation after pediatric cardiac surgery: systematic review, meta-analysis, and evidence-based recommendations. J Card Surg.

[18] Mittnacht AJ, Hollinger I (2010). Fast-tracking in pediatric cardiac surgery - the current standing. Ann Card Anaesth.

